# Lateral Dynamics in Polymer-Supported Membranes

**DOI:** 10.3390/ma5101923

**Published:** 2012-10-19

**Authors:** Shigeyuki Komura, Sanoop Ramachandran, Kazuhiko Seki

**Affiliations:** 1 Department of Chemistry, Graduate School of Science and Engineering, Tokyo Metropolitan University, Tokyo 192-0397, Japan; 2 Polymer Physics Group, Department of Physics, Free University of Brussels, Campus Plaine, CP 223, Brussels 1050, Belgium; E-Mail: sramacha@ulb.ac.be; 3 National Institute of Advanced Industrial Science and Technology (AIST), Ibaraki 305-8565, Japan; E-Mail: k-seki@aist.go.jp

**Keywords:** supported membranes, lipids, viscoelasticity, diffusion, microrheology

## Abstract

We investigate the lateral dynamics in a purely viscous lipid membrane which is supported by a thin polymer sheet (polymer-supported membrane). The generalized frequency-dependent mobility tensor of the polymer-supported membrane is obtained by taking into account the viscoelasticity of the polymer sheet. Due to its viscoelasticity, the cross-correlation functions of two particles embedded in the membrane exhibit an anomalous diffusion. A useful relation for two-point microrheology connecting the cross-correlation function and the modulus of the polymer sheet is provided.

## 1. Introduction

Biomembranes are thin two-dimensional (2D) fluids which separate inner and outer environments of organelles in cells [1]. The fluidity of biomembranes is guaranteed mainly due to the lipid molecules, which are in the liquid crystalline state at physiological temperatures. Proteins and other molecules embedded in biomembranes undergo lateral diffusion, which plays an important role in biological functions [2,3]. In order to study the lateral dynamics in biomembranes, lipid-bilayer membranes supported on solid substrates have been widely used [4]. The standard method is to place membranes, either biomembranes or artificial membranes, directly on solids, which are called as solid-supported membranes. However, experiments using solid-supported membranes have some fundamental difficulties. This is because the membrane-substrate distance is usually not large enough to avoid direct contact between membrane proteins and the solid surface. Such a direct contact leads to a frictional coupling between proteins and the solid support, which may cause protein denaturation.

One way to avoid this problem is to put thin soft polymeric materials between the membrane and the solid substrate [5]. For such polymer-supported membranes, the typical thickness of a polymer cushion is 10–100 nm. Moreover, polymer-supported membranes can be used as cell-surface models that connect biological and artificial materials [6]. In fact, it is known that cytoplasm of eukaryotic cells contains proteins and organelles, including a thick sub-membrane layer of actin-meshwork forming a part of the cell cytoskeleton. Also the extra-cellular fluid consists of extracellular matrix or hyaluronic acid gel. It is important to note that hydrated polymer cushions are generally viscoelastic rather than purely viscous because they are essentially polymer solutions. Recently, it was theoretically shown by us that the mean square displacement of a single inclusion embedded in a viscous membrane exhibits an anomalous diffusion due to the viscoelasticity of the surrounding solvent [7]. This means that the presence of a polymer sheet could have a significant influence on the membrane lateral dynamics.

In this paper, we discuss the dynamics and response of polymer-supported membranes by taking into account the viscoelastic property of the thin polymer sheet. Focusing on the two-particle tracking case, we show that the viscoelasticity of the polymer cushion leads to an anomalous (subdiffusive) dynamics in the 2D viscous membrane. We also give a relation connecting the cross-correlation function and the modulus of the polymer sheet, which is useful for two-point microrheology experiments.

In the next section, we shall first discuss the hydrodynamic mobility tensor of polymer-supported membranes. Using this mobility tensor, we obtain in Section 3 the cross-correlation functions of two distinct particle positions, and argue the effects due to the viscoelasticity of the ambient polymer sheet. In Section 4, we give a useful relation for two-point microrheology as well as some discussion.

## 2. Mobility Tensor of Polymer-Supported Membranes

**Figure 1:**
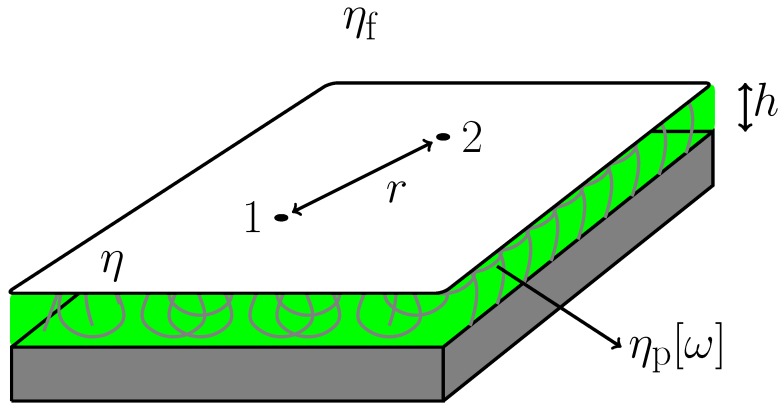
Schematic picture showing a planar viscous membrane with 2D viscosity *η*. It is supported by a viscoelastic polymer cushion of 3D frequency-dependent viscosity $\eta_{p}\left[\omega \right]$. The distance between the membrane and the solid substrate (or the thickness of the polymer sheet) is *h*. The upper region of the film is occupied by a semi-infinite fluid with 3D viscosity $\eta_{f}$. The two correlated point particles “1" and “2" are separated by a distance *r*.

Our model system is similar to those in [7,8,9] and is schematically depicted in Figure 1. A flat thin film of purely viscous membrane with 2D viscosity *η* lies on the xy-plane a distance *h* away from a solid substrate. The space between the thin film and the substrate is filled with a viscoelastic polymer sheet with frequency dependent 3D viscosity $\eta_{p}\left[\omega \right]$. The space on the other side of the film is occupied by a semi-infinite fluid with 3D viscosity $\eta_{f}$. All fluids including the membrane are assumed to be incompressible and their inertial effects are neglected here. The latter assumption is always justified as long as we are concerned with a long-time behavior.

A convenient way to characterize the membrane dynamics as a fluid medium is through the hydrodynamic mobility tensor G, which relates the membrane velocity v to a point force F. Within the linearized hydrodynamics, they are related by

(1) v[k,ω]=G[k,ω]·F[k,ω].

Here the 2D Fourier transform in space and the Fourier–Laplace transform in time for any function of space and time f(r,t) is defined by $f\left[k,\omega \right]=\int_{-\infty }^{\infty }d^{2}r\;\int_{0}^{\infty }dt\;f\left(r,t\right)\exp\left[-i\left(k\cdot r+\omega t\right)\right]$, where $k=(k_{x},k_{y})$ is the 2D wavevector and *ω* the angular frequency. The mobility tensor for a supported membrane is given by the Equation (80) in [9] when the surrounding fluids are purely viscous, and how to take into account the viscoelasticity of the ambient fluid is discussed in [7]. Using these previous results, we obtain the frequency-dependent mobility tensor for a polymer-supported membrane (see Figure 1) as

(2) $$G_{\alpha \beta }\left[k,\omega \right]=\frac{1}{\eta k^{2}+\eta_{f}k+\eta_{p}\left[\omega \right]k\coth\left(kh\right)}\left(\delta_{\alpha \beta }-\frac{k_{\alpha }k_{\beta }}{k^{2}}\right),$$

where α,β=x,y and k=|k|. We note that the case when h→∞ and $\eta_{p}\left[\omega \right]=\eta_{f}$ (without polymer) was originally considered by Saffman and Delbrück [10,11,12].

Since we are interested in polymer-supported membranes, we follow here the discussion in [13], and consider the limit of a vanishingly small h→0, as opposed to the Saffman–Delbrück case. Then the above expression is approximated as

(3) $$G_{\alpha \beta }\left[k,\omega \right]\approx \frac{1}{\eta (k^{2}+\nu k+\kappa^{2}\left[\omega \right])}\left(\delta_{\alpha \beta }-\frac{k_{\alpha }k_{\beta }}{k^{2}}\right),$$

where $\nu =\eta_{f}/\eta$ and

(4) $$\kappa \left[\omega \right]={\left(\frac{\eta_{p}\left[\omega \right]}{\eta h}\right)}^{1/2}.$$

Notice that κ[ω] does not depend on *k*. Equation (3) has two poles at $k_{\pm }=\nu \left[-1\pm \sqrt{1-4{\left(\kappa /\nu \right)}^{2}}\right]/2$, which in the limit h→0 turn into $k_{\pm }=\pm i\kappa$. Hence, for sufficiently small *h*, the mobility tensor takes the form of

(5) $$G_{\alpha \beta }\left[k,\omega \right]\approx \frac{1}{\eta (k^{2}+\kappa^{2}\left[\omega \right])}\left(\delta_{\alpha \beta }-\frac{k_{\alpha }k_{\beta }}{k^{2}}\right).$$

This expression indicates that the presence of the upper viscous fluid becomes irrelevant in the limit of h→0. In the limit of ω→0, Equation (5) reduces to the static mobility tensor discussed by Evans and Sackmann [14,15] and later generalized by us [16]. In the following, we shall use Equation (5) for the frequency-dependent mobility tensor of polymer-supported membranes.

Concerning the viscoelasticity of the hydrated polymer sheet, we assume that its complex modulus obeys a power-law behavior $G_{p}\left[\omega \right]=G_{0}{\left(i\omega \right)}^{\alpha }$ with α<1, as generally argued by Granek [17]. This behavior is commonly observed for various polymeric solutions at high frequencies. Examples are α=1/2 and α=2/3 for Rouse and Zimm dynamics, respectively [18], and α=3/4 for semi-dilute solutions of semi-flexible polymers such as actin filaments [19]. For particle-tracking experiments [20,21], it is more convenient to work in the Laplace domain defined by $\tilde{f}\left(s\right)=\int_{0}^{\infty }dt\;f\left(t\right)e^{-st}$. One can easily convert from the Fourier–Laplace domain to the Laplace domain by substituting s=iω. Hence ${\tilde{G}}_{p}\left(s\right)=G_{0}s^{\alpha }$, and the Laplace transform of the polymer sheet viscosity is given by ${\tilde{\eta }}_{p}\left(s\right)={\tilde{G}}_{p}\left(s\right)/s=G_{0}s^{\alpha -1}$. Therefore Equation (4) can be written in the Laplace domain as

(6) $$\tilde{\kappa }\left(s\right)={\left(\frac{G_{0}s^{\alpha -1}}{\eta h}\right)}^{1/2}.$$

## 3. Two-Particle Correlated Dynamics

Using Equation (5) and Equation (6), we have discussed in [7] the Brownian motion of a circular disk immersed in a membrane. Here we discuss the correlated dynamics of two distinctive particles immersed in a polymer supported membrane. This situation is relevant to two-point microrheology experiments [22] as further discussed in the next section. Compared to single-particle microrheology, there are several advantages to perform multi-particle microrheology [21]. For example, long-time convective drift can be automatically subtracted in this method so that measurements of probe self-diffusivities become possible over longer times. Multi-particle techniques can be also used to investigate heterogeneous materials.

Consider a pair of point particles embedded in the membrane undergoing Brownian motion separated by a 2D vector r. The quantity of interest is the cross-correlation function (CCF) of the particle displacements ${\langle \Delta r_{\alpha }^{1}\left(0\right)\Delta r_{\beta }^{2}\left(t\right)\rangle }_{r}$, where $\Delta r_{\alpha }^{i}$ is the displacement of the particle *i* (=1,2) along the axis *α* (=x,y). We also define the *x*-axis to be along the line connecting the two particles, *i.e.*, $r=r{\hat{e}}_{x}$. According to the fluctuation dissipation theorem, the CCF is related to the time-dependent coupling mobility $M_{\alpha \beta }\left(t\right)$ in the Laplace domain as [21]

(7) $${\langle \Delta {\tilde{r}}_{\alpha }^{1}\Delta {\tilde{r}}_{\beta }^{2}\left(s\right)\rangle }_{r}=\frac{2k_{B}T}{s^{2}}{\tilde{M}}_{\alpha \beta }\left(r,s\right),$$

for sufficiently large *r*. The inverse Laplace transform of Equation (7) provides us with the time-dependent CCF:

(8) $${\langle \Delta r_{\alpha }^{1}\left(0\right)\Delta r_{\beta }^{2}\left(t\right)\rangle }_{r}=\frac{1}{2\pi i}\int_{c-i\infty }^{c+i\infty }ds\;\frac{2k_{B}T}{s^{2}}{\tilde{M}}_{\alpha \beta }\left(r,s\right)e^{st}.$$

Since $M_{xy}=0$ by symmetry, it is sufficient to consider the longitudinal coupling mobility $M_{xx}$ and the transverse one $M_{yy}$.

Notice that the coupling mobility $M_{\alpha \beta }\left(r\right)$ is directly related to the inverse Fourier transform (in space) of the mobility tensor $G_{\alpha \beta }\left(r\right)$. Since $G_{\alpha \beta }\left(r\right)$ can generally be expressed as $G_{\alpha \beta }\left(r\right)=C_{1}\left(r\right)\delta_{\alpha \beta }+C_{2}\left(r\right)r_{\alpha }r_{\beta }/r^{2}$, the longitudinal and the transverse coupling mobilities are given by $M_{xx}\left(r\right)=C_{1}\left(r\right)+C_{2}\left(r\right)$ and $M_{yy}\left(r\right)=C_{1}\left(r\right)$, respectively.

### 3.1. Longitudinal Coupling

**Figure 2:**
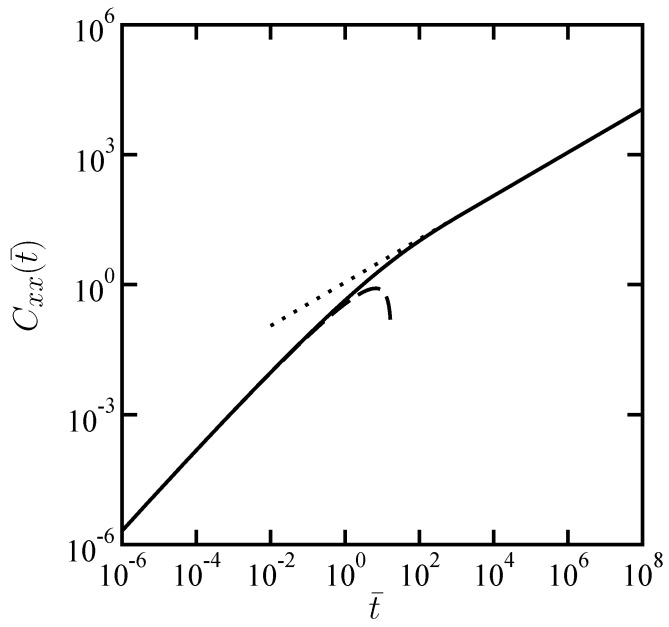
Scaled longitudinal cross-correlation function $C_{xx}\left(\bar{t}\right)$ as a function of scaled time $\bar{t}$ when α=1/2. These dimensionless quantities are defined in the text. The dotted and dashed lines correspond to the asymptotic expressions given by Equation (11) and Equation (13), respectively.

By utilizing Equation (5) and the results in References [9,13,23], the coupling mobilities for polymer-supported membranes can be obtained analytically. First the Laplace transform of the longitudinal coupling mobility is given by

(9) $${\tilde{M}}_{xx}\left(r,s\right)=\frac{1}{2\pi \eta }\left[\frac{1}{{\left(\tilde{\kappa }\left(s\right)r\right)}^{2}}-\frac{K_{1}\left[\tilde{\kappa }\left(s\right)r\right]}{\tilde{\kappa }\left(s\right)r}\right],$$

where $K_{1}\left[z\right]$ is the modified Bessel function of the second kind, order one. In Figure 2, we plot the result of numerical inverse Laplace transform of Equation (8) with the longitudinal coupling mobility Equation (9) when α=1/2 corresponding to the Rouse dynamics. Here the dimensionless CCF and time are defined by $C_{xx}\left(\bar{t}\right)=\left(\pi \eta /k_{B}T\right){\left(\eta h/G_{0}r^{2}\right)}^{1/(\alpha -1)}{\langle \Delta r_{\alpha }^{1}\left(0\right)\Delta r_{\beta }^{2}\left(t\right)\rangle }_{r}$ and $\bar{t}={\left(\eta h/G_{0}r^{2}\right)}^{1/(\alpha -1)}t$, respectively.

In the limit of a large distance r→∞ ($\tilde{\kappa }\left(s\right)r\gg 1$), the above expression can be approximated as

(10) $${\tilde{M}}_{xx}\left(r,s\right)\approx \frac{1}{2\pi \eta }\frac{1}{{\left(\tilde{\kappa }\left(s\right)r\right)}^{2}}.$$

When the viscosity of the polymer cushion obeys the power-law behavior ${\tilde{\eta }}_{p}\left(s\right)=G_{0}s^{\alpha -1}$, as assumed in the previous section, we use Equation (6) for $\tilde{\kappa }\left(s\right)$ and perform the inverse Laplace transform given in Equation (8). Then we obtain

(11) $${\langle \Delta r_{x}^{1}\left(0\right)\Delta r_{x}^{2}\left(t\right)\rangle }_{r}\approx \frac{k_{B}Th}{\pi G_{0}\Gamma \left[1+\alpha \right]}\frac{t^{\alpha }}{r^{2}},$$

where Γ[z] is the gamma function. This result shows that the viscoelasticity of the polymer sheet leads to a subdiffusive time dependence of the CCF. Since α<1, the viscoelasticity slows down the normal diffusion process. The $1/r^{2}$-dependence in Equation (11) arises from the mass conservation in 2D rather than the momentum conservation [24].

In the limit of a small distance r→0 ($\tilde{\kappa }\left(s\right)r\ll 1$), on the other hand, Equation (9) asymptotically behaves as

(12) $${\tilde{M}}_{xx}\left(r,s\right)\approx \frac{1}{4\pi \eta }\left[\ln\left(\frac{2}{\tilde{\kappa }\left(s\right)r}\right)-\gamma +\frac{1}{2}\right],$$

where γ=0.5772⋯ is the Euler’s constant. Following the same process as above, the longitudinal CCF asymptotically behaves as

(13) $${\langle \Delta r_{x}^{1}\left(0\right)\Delta r_{x}^{2}\left(t\right)\rangle }_{r}\approx \frac{k_{B}T}{4\pi \eta }t\left[\ln\left(\frac{4\eta ht^{\alpha -1}}{G_{0}r^{2}}\right)+\left(\alpha -3\right)\gamma -\alpha +2\right],$$

which grows like tln(1/t). Such a logarithmic correction leads to a time-dependent diffusivity. It should be noted, however, that this asymptotic expression is valid only when $G_{0}r^{2}t^{1-\alpha }/\eta h\ll 1$. The asymptotic expressions given by Equation (11) and Equation (13) are also plotted in Figure 2, which shows an agreement with the numerical evaluation.

### 3.2. Transverse Coupling

**Figure 3:**
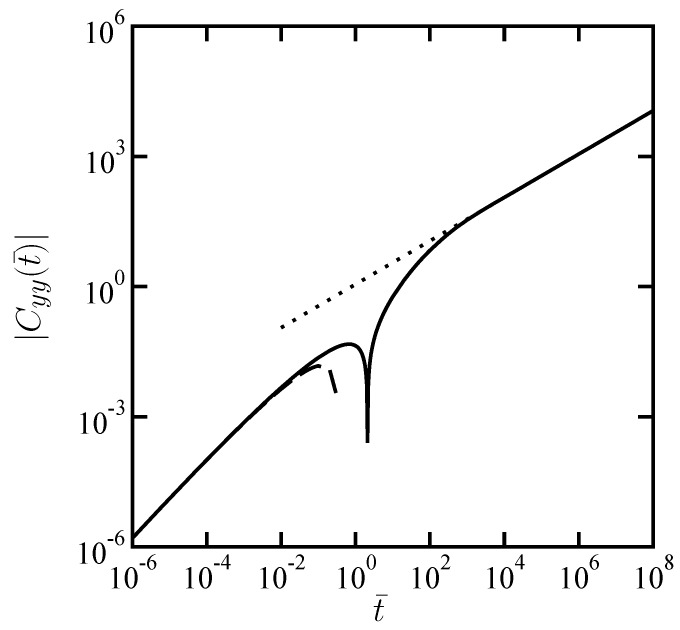
Scaled transverse cross-correlation function $|C_{yy}\left(\bar{t}\right)|$ as a function of scaled time $\bar{t}$ when α=1/2. Notice that $C_{yy}\left(\bar{t}\right)$ can be negative. The dotted and dashed lines correspond to the asymptotic expressions given by Equation (16) and Equation (18), respectively.

Next we discuss the transverse coupling motion. The transverse coupling mobility is analytically given by [9,13,23]

(14) $${\tilde{M}}_{yy}\left(r,s\right)=\frac{1}{2\pi \eta }\left[K_{0}\left[\tilde{\kappa }\left(s\right)r\right]+\frac{K_{1}\left[\tilde{\kappa }\left(s\right)r\right]}{\tilde{\kappa }\left(s\right)r}-\frac{1}{{\left(\tilde{\kappa }\left(s\right)r\right)}^{2}}\right],$$

where the $K_{0}\left[z\right]$ is the modified Bessel functions of the second kind, order zero. The numerically evaluated dimensionless transverse CCF is shown in Figure 3. Since the transverse CCF becomes negative for large $\bar{t}$, we have taken its absolute values in the log-log plot.

In the large distance limit of r→∞, Equation (14) asymptotically behaves as

(15) $${\tilde{M}}_{yy}\left(r,s\right)\approx -\frac{1}{2\pi \eta }\frac{1}{{\left(\tilde{\kappa }\left(s\right)r\right)}^{2}},$$

which is indeed negative. Hence the time dependence of transverse CCF is simply given by

(16) $${\langle \Delta r_{y}^{1}\left(0\right)\Delta r_{y}^{2}\left(t\right)\rangle }_{r}\approx -\frac{k_{B}Th}{\pi G_{0}\Gamma \left[1+\alpha \right]}\frac{t^{\alpha }}{r^{2}}.$$

In the small distance limit of r→0, on the other hand, Equation (14) becomes

(17) $${\tilde{M}}_{yy}\left(r,s\right)\approx \frac{1}{4\pi \eta }\left[\ln\left(\frac{2}{\tilde{\kappa }\left(s\right)r}\right)-\gamma -\frac{1}{2}\right],$$

and its inverse Laplace transform yields the following transverse CCF

(18) $${\langle \Delta r_{y}^{1}\left(0\right)\Delta r_{y}^{2}\left(t\right)\rangle }_{r}\approx \frac{k_{B}T}{4\pi \eta }t\left[\ln\left(\frac{4\eta ht^{\alpha -1}}{G_{0}r^{2}}\right)+\left(\alpha -3\right)\gamma -\alpha \right].$$

Similarly as before, the asymptotic expressions in Equation (16) and Equation (18) are also plotted in Figure 3.

## 4. Discussion

In this paper, we have discussed the lateral dynamics in a purely viscous lipid membrane supported by a viscoelastic polymer sheet. Using the generalized frequency-dependent mobility tensor for the polymer supported membrane, we obtained the cross-correlation function (CCF) of two point particles embedded in the membrane. The obtained CCF exhibits a subdiffusive time dependence reflecting the viscoelastic property of the polymer cushion.

Since the subdiffusive behavior is expected for $\bar{t}\gg 1$, we shall estimate the value of the crossover time $t^{*}$ from the condition $\bar{t}∼1$. Here we use the values $\eta ∼10^{-10}$ Pa·s·m [25], $h∼10^{-7}$ m [6], and $r∼10^{-6}$ m. Some consideration is required for the value of $G_{0}$ because its dimension depends on the value of *α* due to our definition $G_{p}\left[\omega \right]=G_{0}{\left(i\omega \right)}^{\alpha }$. Following the description of polymer solutions in [18], we expect that $G_{0}$ roughly scales as $G_{0}∼G\tau^{\alpha }∼G{\left(\eta_{s}/G\right)}^{\alpha }∼G^{1-\alpha }\eta_{s}^{\alpha }$ where *G* is the constant modulus, *τ* is the slowest relaxation time, and $\eta_{s}$ is the solvent viscosity. Using the values G∼10 Pa [26] and $\eta_{s}∼10^{-3}$ Pa·s, the crossover time is roughly estimated to be $t^{*}∼10^{-8}$ s when α=1/2. Since this crossover time is rather short, one would observe the subdiffusive behavior in most of the realistic experiments such as microrheology. When *r* is of the order of a protein size, say $r∼10^{-8}$ m, the crossover time is much longer and becomes $t^{*}∼1$ s. In this case, the lateral dynamics is almost diffusive up to $t^{*}$. However, the subdiffusive behavior becomes always dominant when t→∞.

So far, we have assumed that the viscoelasticity of the polymer sheet obeys a simple power-law behavior, *i.e.*, $G_{p}\left[\omega \right]=G_{0}{\left(i\omega \right)}^{\alpha }$. This is certainly an oversimplified assumption, and the general frequency dependence of the polymer solution is more complex [18]. In fact, the technique of microrheology enables us to measure the local mechanical response of various soft matter over a wide frequency range [27,28]. For two-point microrheology, the measurement of the CCF gives the rheological information of the hydrated polymer cushion. This can be most easily demonstrated by the large distance behavior of the longitudinal coupling mobility given by Equation (10). With the use of Equation (7), we have

(19) $${\langle \Delta {\tilde{r}}_{x}^{1}\Delta {\tilde{r}}_{x}^{2}\left(s\right)\rangle }_{r}=\frac{k_{B}Th}{\pi r^{2}s^{2}{\tilde{\eta }}_{p}\left(s\right)}=\frac{k_{B}Th}{\pi r^{2}s{\tilde{G}}_{p}\left(s\right)}.$$

This equation relates the observed transverse CCF to the modulus of the underlying polymer sheet (rather than the membrane). In other words, we can extract the 3D bulk information by using the 2D dynamics taking place in the membrane. Once ${\tilde{G}}_{p}\left(s\right)$ is obtained from the experiment, the frequency dependence of the storage and the loss moduli can be deduced by identifying $G_{p}\left[\omega \right]=G_{p}^{\prime }\left[\omega \right]+iG_{p}^{\prime \prime }\left[\omega \right]={\tilde{G}}_{s}\left(s=i\omega \right)$. Notice that these two representations are equivalent because $G_{p}^{\prime }\left[\omega \right]$ and $G_{p}^{\prime \prime }\left[\omega \right]$ are related by the Kramers–Kronig relation [20,21].

Some caution is required when applying our theory to experiments. For two-particle tracking, the distance between the two point particles (which can be small membrane proteins) should be larger than the network mesh size. Otherwise, the viscoelastic effect is not reflected in the lateral dynamics. In order to obtain the full time behavior of the particle motion, one should use a viscoelastic modulus that is dependent both on wavevector and frequency.

In this paper, we have intentionally treated the membrane as a purely viscous 2D fluid in order to emphasize the role of the ambient polymer sheet. In general, however, lipid membranes themselves can also be viscoelastic. Recently, viscoelasticity of phospholipid Langmuir monolayers in a liquid-condensed phase was measured using active microrheology [29,30]. The viscoelasticity of membrane itself was theoretically taken into account in [31,32].
